# RSR13, an allosteric effector of haemoglobin, and carbogen radiosensitize FSAII and SCCVII tumours in C3H mice

**DOI:** 10.1038/sj.bjc.6690130

**Published:** 1999-02

**Authors:** S R Khandelwal, B D Kavanagh, P-S Lin, Q T Truong, J Lu, D J Abraham*, R K Schmidt-Ullrich

**Affiliations:** Departments of Radiation Oncology and Medicinal Chemistry*, Medical College of Virginia, Richmond, VA, 23298, USA

**Keywords:** RSR13, haemoglobin–oxygen affinity, allosteric effectors of haemoglobin, tumour hypoxia, radiosensitizer

## Abstract

Pre-clinical evaluation has demonstrated that 2-[4-(((3,5-dimethylanilino)carbonyl)methyl)phenoxy]-2-methylpropionic acid (RSR13) acts as an allosteric effector of haemoglobin (Hb). RSR13 binding to Hb results in decreased haemoglobin–oxygen (Hb–O_2_) affinity, improved tumour oxygenation, and enhanced radiation-induced cell killing in several experimental tumour systems. In the present work, ex vivo clonogenic survival analyses are applied in two murine tumour systems to characterize the relationship between the magnitude of decrease in Hb–O_2_ affinity and radiosensitization, the influence of inspired *p*O_2_ upon this effect, and the efficacy of combining RSR13 and radiation during a course of repeated radiation exposures. For FSaII tumours in C3H mice breathing air, 100 mg kg^−1^ RSR13 administered intraperitoneally produced an enhancement ratio (ER) of 1.3, but there was marked desensitization at a RSR13 dose of 300 mg kg^−1^ (ER 0.6). The most likely reason for the increased radioresistance was insufficient oxygen loading of Hb in the pulmonary circulation due to reduced haemoglobin–oxygen affinity because carbogen breathing combined with 300 mg kg^−1^ RSR13 reversed the effect and produced an ER of 1.8. In SCCVII tumours in C3H mice irradiated with eight fractions of 2.5 Gy over 4 days, the surviving fraction was reduced to 58–67% of control values when RSR13 was combined with radiation on days 1 and 2, days 3 and 4, or days 1–4. These results confirm that combining RSR13 and irradiation within a fractionated course of clinically relevant low-dose exposures provides significant radiosensitization. Additional preclinical experimentation is needed to define better the optimum dose-scheduling conditions for clinical applications. *1999 Cancer Research Campaign*


*British Journal of Cancer* (1999) **79**(5/6), 814–820
© 1999 Cancer Research Campaign
Article no. bjoc.1998.0130

### RSR13, an allosteric effector of haemoglobin, and carbogen radiosensitize FSAII and SCCVII tumours in C3H mice

**SR Khandelwal, BD Kavanagh, P-S Lin, QT Truong, J Lu, DJ Abraham* and RK Schmidt-Ullrich**

Departments of Radiation Oncology and Medicinal Chemistry*, Medical College of Virginia, Virginia Commonwealth University, Richmond, VA 23298, USA

**Summary** Pre-clinical evaluation has demonstrated that 2-[4-(((3,5-dimethylanilino)carbonyl)methyl)phenoxy]-2-methylpropionic acid (RSR13) acts as an allosteric effector of haemoglobin (Hb). RSR13 binding to Hb results in decreased haemoglobin–oxygen (Hb–$O_2$) affinity, improved tumour oxygenation, and enhanced radiation-induced cell killing in several experimental tumour systems. In the present work, ex vivo clonogenic survival analyses are applied in two murine tumour systems to characterize the relationship between the magnitude of decrease in Hb–$O_2$ affinity and radiosensitization, the influence of inspired $pO_2$ upon this effect, and the efficacy of combining RSR13 and radiation during a course of repeated radiation exposures. For FSaII tumours in C3H mice breathing air, 100 mg kg$^{-1}$ RSR13 administered intraperitoneally produced an enhancement ratio (ER) of 1.3, but there was marked desensitization at a RSR13 dose of 300 mg kg$^{-1}$ (ER 0.6). The most likely reason for the increased radioresistance was insufficient oxygen loading of Hb in the pulmonary circulation due to reduced haemoglobin–oxygen affinity because carbogen breathing combined with 300 mg kg$^{-1}$ RSR13 reversed the effect and produced an ER of 1.8. In SCCVII tumours in C3H mice irradiated with eight fractions of 2.5 Gy over 4 days, the surviving fraction was reduced to 58–67% of control values when RSR13 was combined with radiation on days 1 and 2, days 3 and 4, or days 1–4. These results confirm that combining RSR13 and irradiation within a fractionated course of clinically relevant low-dose exposures provides significant radiosensitization. Additional preclinical experimentation is needed to define better the optimum dose-scheduling conditions for clinical applications.

**Keywords:** RSR13; haemoglobin–oxygen affinity; allosteric effectors of haemoglobin; tumour hypoxia; radiosensitizer

Hypoxia in tumours is recognized as a potential cause for resistance to radiotherapy. The modulation of radiosensitivity by oxygen ($O_2$) is best established for cultured mammalian cells, which are highly resistant to the cytotoxic effects of ionizing radiation under conditions of extremely low partial pressures of oxygen ($pO_2$) of ≤3 mmHg (Hall, 1994). The correlation between tumour control probability and haemoglobin (Hb) concentration also lends indirect evidence to the importance of oxygen delivery as a determinant of radiotherapeutic outcome (Evans and Bergsjo, 1965; Bush et al, 1978; Overgaard et al, 1989). Furthermore, clinical studies involving pretreatment interstitial measurements of $pO_2$ in squamous cell carcinomas of the uterine cervix and the head and neck region have demonstrated an inverse relationship between the extent of tumour hypoxia and tumour control rates after radiotherapy (Gatenby et al, 1988; Hockel et al, 1993; Nordsmark et al, 1996*a*; Brizel et al, 1997).

In an attempt to sensitize tumours to radiotherapy, different techniques for targeting hypoxic cells or improving delivery of oxygen have been used. A meta-analysis of randomized studies involving hypoxic cell radiosensitization via nitroimidazoles revealed significant, albeit small, improvement in local control and survival in patients with head and neck and bladder carcinomas (Overgaard, 1994). The use of hyperbaric oxygen as an adjuvant to radiotherapy for locally advanced cervical cancer was evaluated in several randomized studies, but a benefit was not consistently observed (Fletcher et al, 1977; Ward and Dixon, 1979; Dische, 1983; Brady et al, 1981). An alternative approach to improved oxygen delivery to tumour tissues is to exploit the enormous reservoir of oxygen that remains bound to Hb even after passage of blood through the capillary bed. Pioneering efforts to achieve additional unloading of oxygen from Hb included the use of 2,3-diphosphoglycerol (2,3-DPG) and chlorophenoxy acetic acid derivatives (Siemann et al, 1979; Siemann and Macler, 1986; Hirst and Wood 1987, 1989; Hirst et al, 1987), but did not reach clinical application.

Synthetic allosteric effectors of Hb have now been developed as a new class of pharmaceutical agents for potential therapeutic application in conditions of detrimental tissue hypoxia. One of these compounds, 2-[4-(((3,5-dimethylanilino)carbonyl)methyl)-phenoxy]-2-methylpropionic acid (RSR13) stabilizes Hb in the deoxy state which results in reduced Hb–$O_2$ affinity (Randad et al, 1991; Wireko et al, 1991; Abraham et al, 1992*a*, *b*). This effect of Hb–$O_2$ affinity is quantifiable as an increase in $p50$, defined as the $pO_2$ required for 50% saturation of Hb binding sites. Preclinical evaluation of RSR13 has demonstrated that doses of 100–300 mg kg$^{-1}$ achieve an increase in $p50$ and normal tissue $pO_2$, a decrease in intratumoral hypoxia, and an enhancement of radiation-induced cell killing in several tumour systems (Khandelwal et al, 1993, 1996; Teicher et al, 1996).

*Received 11 March 1998*
*Revised 1 July 1998*
*Accepted 24 July 1998*

*Correspondence to:* RK Schmidt-Ullrich, Department of Radiation Oncology, Medical College of Virginia Box 980058, Richmond, VA 23298-0058, USA

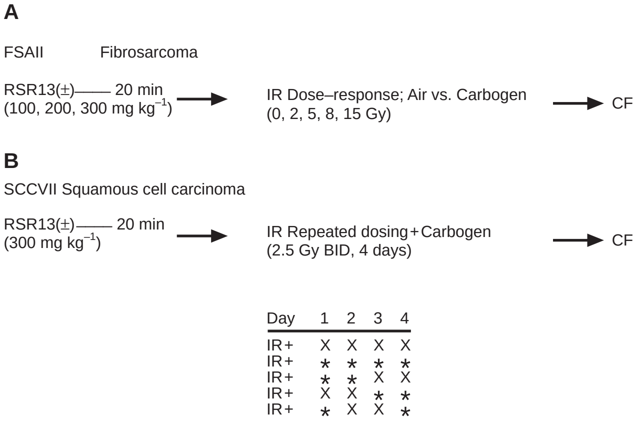

**Figure 1** RSR13/irradiation schedules for clonogenic survival analyses. (**A**) Single-exposure experiments, FSaII tumours. (**B**) Repeated exposure experiments, SCCVII tumours. ×, irradiation alone; *, RSR13 plus irradiation; CF, colony formation; IR, ionizing radiation

To optimize the efficacy of RSR13 combined with radiotherapy, it is important to characterize the quantitative relationship between increases in *p*50 and radiation-induced cytotoxicity and the effect of inspired $pO_2$ upon this effect. It is also essential to establish with greater certainty the enhancement of tumour radiosensitivity within dose schedules that combine RSR13 with repeated radiation exposures, as is typical for a course of fractionated radiotherapy. The present work addresses these issues using highly sensitive ex vivo clonogenic survival analyses in two syngeneic murine tumour systems, both established models of tumour hypoxia (Okunieff et al, 1986; Brown and Lemmon, 1990). For the murine FSaII fibrosarcoma, single-exposure radiation dose–response analyses were generated at different doses of RSR13 and air or carbogen breathing. The schedule dependence of RSR13 combined with repeated radiation exposures in the therapeutic dose range was investigated using SCCVII tumours.

### MATERIALS AND METHODS

### Preparation, dosage and administration of RSR13

RSR13 was supplied by Allos Therapeutics (Denver, CO, USA) as a standardized stock solution of 30 mg $ml^{-1}$ in 0.9% sodium chloride. RSR13 was administered intraperitoneally (i.p.) at doses of 100, 150, 200, 250 and 300 mg $kg^{-1}$. This dose range was based on previous findings that 300 mg $kg^{-1}$ was a well-tolerated dose in C3H mice, increased muscle $pO_2$, and resulted in radiosensitization of FSaII fibrosarcomas (Khandelwal et al, 1993, 1996).

### Animals and tumour models

Male C3H mice were obtained from the Department of Radiation Medicine at the Massachusetts General Hospital (Boston, MA, USA). The maintenance of mice was in compliance with the NIH 1996 regulations for the care and use of laboratory animals. Two syngeneic tumour models in C3H mice were used: the FSaII fibrosarcoma (Okunieff et al, 1986) and the SCCVII squamous cell carcinoma (Brown and Lemmon, 1990; Dorie et al, 1993). FSaII tumour cells were handled as described previously (Khandelwal et al, 1996). For SCCVII tumours, original tumour cell stocks and protocols for tumour maintenance were kindly provided by Dr JM Brown. Estimates of the hypoxic fraction of SCCVII tumours have ranged from 1% to 20%, and this tumour has been previously used in experiments involving fractionated radiation exposures (Horsman et al, 1994; Brown and Lemmon, 1990). The percentage of radiobiological hypoxia within FSaII tumours of a size similar to those used in the present experiments has been reported to be in the range of 12–17% (Rice et al, 1980; Gerweck et al, 1992).

Both tumour types were propagated by identical methodology. Briefly, FSaII/SCCVII cells were injected into C3H mice to establish tumours from which single-cell suspensions were generated for frozen stocks. These stocks were used to maintain relatively short-term cultures for up to four passages. From each in vitro passage, tumours were propagated in vivo by subcutaneous injection of $7.5 \times 10^3$ FSaII cells or $3 \times 10^4$ SCCVII cells into the right hind leg of C3H mice.

### Effects of RSR13 on $Hb–O_2$ affinity and the oxygen equilibrium curve

For the tonometry analyses described below, changes in $Hb–O_2$ affinity were determined from blood samples obtained at 20, 40 and 60 min after RSR13 administration. Results were displayed in the form of oxygen equilibrium curves (OEC) in which $pO_2$ was plotted against per cent of total oxygen saturation of $Hb(SO_2)$. The reduction of $Hb–O_2$ affinity was characterized by a right-hand shift of the OEC and corresponds to an increase in *p*50. As controls for RSR13 administration, mice were injected with identical volumes of 0.9% sodium chloride.

For the ex vivo tonometry, RSR13 at doses of 100, 150, 200, 250 and 300 mg $kg^{-1}$ was injected i.p. into mice to establish the time course and dose–response data for the increase in *p*50. Between 20 and 60 min after administration of RSR13, the mice were killed by carbon dioxide asphyxiation, and an average of 0.3 ml of blood was collected from each mouse by cardiac puncture. For each RSR13 dose and time point, heparinized blood samples from six mice were pooled for multipoint tonometry.

Tonometry was performed using an automated blood gas analyser and co-oximeter (Instrumentation Laboratories, Lexington, MA, USA) against gas mixtures containing varied oxygen concentrations of 2.95%, 5.85% and 8.75%, and a fixed carbon dioxide concentration of 5.8% with a balance of nitrogen. All samples were incubated at 37°C for 10 min, and Hb concentration and pH values were determined. The percent Hb saturation with oxygen ($SO_2$) was measured by co-oximetry. The OEC data was fitted to a standard Hill's equation curve by non-linear regression analyses using Scientist software (MicroMath, Salt Lake City, UT, USA).

### Radiation procedures

FSaII or SCCVII tumours were studied when the greatest tumour diameter reached 6.5–8 mm. Non-anaesthetized, unrestrained tumour-bearing mice received single, whole-body radiation exposures at a dose rate of 1.7 Gy $min^{-1}$ using a $^{60}Co$ teletherapy unit. Control animals were sham irradiated. Irradiation was administered 20 min after i.p. injection of RSR13 or an identical volume of 0.9% sodium chloride as control. Mice were killed by carbon dioxide asphyxiation 18 h after administration of the assigned treatment.

*© Cancer Research Campaign 1999*

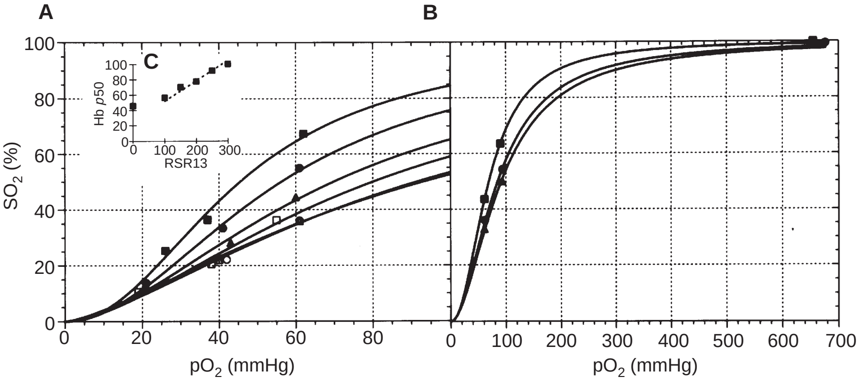

**Figure 2** Dose-dependent effects of RSR13 on Hb–$O_2$ affinity. (**A**) The OECs 20 min after i.p. administration of RSR13 to C3H mice. The curves shift to the right as the Hb–$O_2$ affinity decreases. RSR13 dose: 0 mg $kg^{-1}$ (■); 100 mg $kg^{-1}$ (●); 150 mg $kg^{-1}$ (▲); 200 mg $kg^{-1}$ (□); 250 mg $kg^{-1}$ (○); 300 mg $kg^{-1}$ (△). The 250 mg $kg^{-1}$ and 300 mg $kg^{-1}$ curves are nearly superimposed. (**B**) $SO_2$ was measured at 60, 90 and ~700 mmHg 20 min after i.p. administration of RSR13 to C3H mice. The lower values of $SO_2$ observed at arterial $pO_2$ (~100 mmHg) in Figure 2A were increased to near-complete saturation at $pO_2$ of ~700 mmHg. RSR13 dose: 200 mg $kg^{-1}$ (■); 250 mg $kg^{-1}$ (●), 300 mg $kg^{-1}$ (▲). (**C**) The $pO_2$ at which Hb is 50% saturated with oxygen (*p*50) 20 min after RSR13 administration. The RSR13 dose–response is approximately linear in this range

### Clonogenic survival after RSR13-mediated radiosensitization

The radiosensitization of tumours by RSR13 was quantified using ex vivo clonogenic survival analyses addressing different aspects with each of the two tumour systems. FSaII tumours were used to correlate RSR13 dosing with increases in *p*50 and the relative radiosensitization under conditions of air and carbogen breathing. In these experiments, mice bearing FSaII tumours received i.p. injections of saline or RSR13 at doses of 100, 200 or 300 mg $kg^{-1}$ 20 min before irradiation with single exposures in the dose range of 5–15 Gy. The animals breathed air or carbogen 5 min before and during irradiation (see below). All data points were obtained using a minimum of four animals per combination of radiation dose, RSR13 dose, and inspiratory gas.

SCCVII tumours were used to evaluate the impact of varying the exposure schedule of RSR13 and radiation during a course of repeated radiation exposures simulating a fractionated course of radiotherapy. Mice bearing SCCVII tumours breathed carbogen starting 5 min before and during the time of irradiation. The animals were treated with eight exposures of 2.5 Gy given twice daily for 4 days with a minimum 6-h interval between the two daily radiation exposures. RSR13, 300 mg $kg^{-1}$, was injected i.p. 20 min before each radiation exposure following a schedule depicted in Figure 1.

At the time of ex vivo clonogenic survival analysis, SCCVII tumours were excised and cut into small pieces. A pronase/DNAase/collagenase mixture (Brown and Lemmon, 1990; Dorie et al, 1993) was used to prepare single-cell suspensions. Cells were plated in Waymouth's medium supplemented with 15% fetal bovine serum. Colonies of 50 or more cells were counted 10 days after plating. FSaII tumours were assayed for clonogenic survival as previously described (Khandelwal et al, 1993, 1996). For both tumour types, the data obtained from an individual animal tumour represents the average value of clonogenic assay performed in quadruplicate.

### Clonogenic survival statistical analyses

For FSaII tumours, linear regression was used to analyse the logarithm of clonogenic survival after doses of 5–15 Gy. The enhancement ratio (ER) for each treatment group was defined as follows:

$$\text{ER} = \frac{\text{radiation dose without RSR13 for 10\% surviving fraction}}{\text{radiation dose with RSR13 for 10\% surviving fraction}}$$

A *t*-test was used for comparisons of the logarithms of surviving fractions of SCCVII tumour cell after varying schedules of RSR13 administration. However, because multiple applications of that test at the significance level of 0.05 can increase the probability of a type I error, both the Newman–Kuels and Tukey methods of multiple data set comparisons were also used to test for significant differences between pairs of RSR13–radiation dose schedules. Curves fitted by linear regression are compared by analysing the confidence interval of the estimated slopes and intercepts. All statistical analyses of clonogenic survival data were performed with GraphPad Prism (GraphPad Software, San Diego, CA, USA).

### RESULTS

### Oxygen equilibrium curve (OEC)

The in vivo RSR13 dose–response data for Hb–$O_2$ affinity are illustrated by OECs in which the abscissa is $pO_2$ and the ordinate represents $SO_2$ (Figure 2A and B). The pH and Hb concentrations of the blood samples were nearly constant and did not significantly affect the multipoint tonometry measurements. In the pooled heparinized blood samples, the ranges (±s.d.) of pH measurements and Hb concentrations were 7.14 (±0.03)–7.20 (±0.03) and 12.3 (±0.15)–13.0 (±0.7) g $dl^{-1}$ respectively. The right-hand shift of OEC as a function of time after RSR13 administration of 200 and 300 mg $kg^{-1}$ was initially examined at 20, 40 and 60 min. The maximum right-hand shift was observed 20 min after i.p. administration of RSR13 with diminishing effects at 40 and 60 min (data not shown). Because of this finding, all blood samples for tonometric analysis were obtained 20 min after i.p. administration of RSR13. Figure 2A illustrates the fitted OECs based on $SO_2$ values determined at $pO_2$ values of 20, 40 and 60 mmHg after RSR13 doses of 100, 150, 200, 250 or 300 mg $kg^{-1}$. The initial OECs (Figure 2A) indicated that after RSR13 doses of 200–300 mg $kg^{-1}$ $SO_2$ remained below 60% at a $pO_2$ of 100 mmHg, which corresponds to arterial $pO_2$ during air breathing. To confirm that conditions of higher $pO_2$ provided $SO_2$ values consistent with near-complete Hb loading, the multipoint tonometry conditions

© Cancer Research Campaign 1999

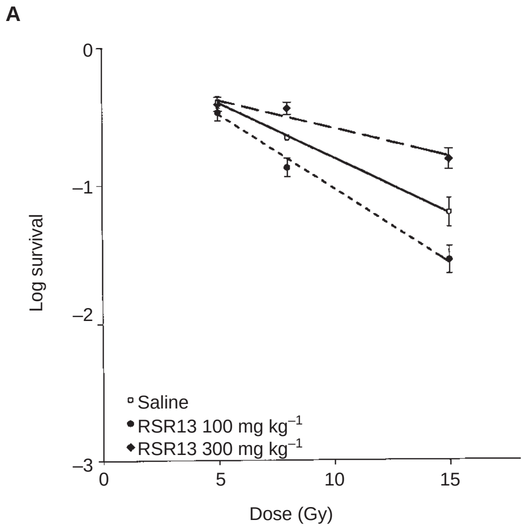

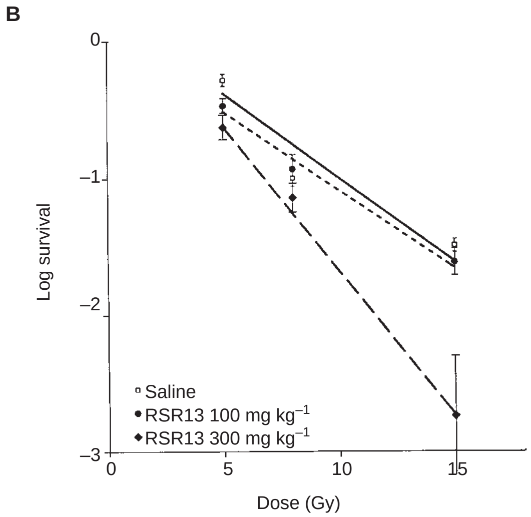

**Figure 3** The effects of RSR13, air or carbogen breathing, and radiation dose on FSaII cell survival. Regression analysis was used to fit the clonogenic survival data for doses of 5, 8 and 15 Gy. Data points are the means ± s.e.m. from a minimum of four animals per condition. Solid line = saline; dotted line = RSR13 100 mg kg$^{-1}$; dashed line = RSR13 300 mg kg$^{-1}$. (**A**) Air breathing; (**B**) carbogen breathing

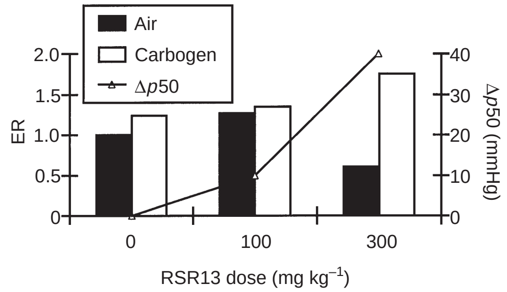

**Figure 4** Relationship between RSR13 dose and increase in *p*50 and enhancement ratio (ER) for FsaII fibrosarcoma cells. The horizontal axis indicates RSR13 dose. Columns indicate the ER after air breathing (black) or carbogen breathing (white), with values indicated on the left vertical axis. On the right vertical axis are values of increase in *p*50; Δ*p*50, represented graphically as a solid line were modified to measure $SO_2$ at $pO_2$ values of 60, 90 and approximately 700 mmHg, corresponding to the $pO_2$ of carbogen. Figure 2B shows that after RSR13 doses of 200–300 mg kg$^{-1}$ $SO_2$ approached 100% when Hb was exposed to carbogen. Thus, carbogen breathing would provide effective Hb loading even with markedly reduced Hb–$O_2$ affinity. To quantify the right-hand shift of the OEC for each RSR13 dose, the *p*50 values were calculated and plotted over the RSR13 dose range used (Figure 2C, inset). The *p*50 values demonstrated an approximately linear dose–response in the range between 100 and 300 mg kg$^{-1}$ RSR13 (Figure 2C).

### RSR13-induced radiosensitization of tumours

The effect of RSR13 on the radiosensitivity of hypoxic FSaII fibrosarcomas was quantified by clonogenic survival. The ex vivo survival analyses after single radiation exposures under conditions of air and carbogen breathing of the mice are shown in Figure 3A and B respectively. The radiation survival curves shown have been generated after in vivo irradiation and administration of saline or RSR13 at doses of 100 or 300 mg kg$^{-1}$. Linear regression analyses demonstrate that RSR13 at 100 mg kg$^{-1}$ and air breathing resulted in significant radiosensitization ($P = 0.01$); this effect differs from the marked decrease in radiosensitivity at 300 mg kg$^{-1}$ of RSR13, under which condition the oxygen loading of Hb in the pulmonary circulation could be compromised because of the expected large reduction in Hb–$O_2$ affinity. Figure 3B illustrates that this apparently insufficient oxygen loading of Hb could be overcome by carbogen breathing, as indicated by the finding that RSR13 at the dose of 300 mg kg$^{-1}$ combined with carbogen breathing resulted in the most significant enhancement of radiation cytotoxicity relative to all other treatment conditions ($P = 0.0001$) including carbogen alone ($P = 0.009$). The calculated ER values and the surviving fraction (SF) of cells relative to control conditions are listed in Table 1.

The effects of different RSR13 doses on increases in *p*50 and ER under conditions of air or carbogen breathing are plotted in Figure 4. It can be seen that the ER continues to increase with higher RSR13 doses under conditions of carbogen breathing but not with air breathing. Although the ER for 300 mg kg$^{-1}$ carbogen rises to 1.8 at a *p*50 of 40 mmHg, there is an actual diminution of radiation-induced killing at the highest dose of RSR13 with air breathing.

### Relative RSR13 effects during repeated radiation exposures

The potential radiosensitizing effects of RSR13 after smaller repeated radiation exposures was examined under conditions of 300 mg kg$^{-1}$ RSR13 and carbogen breathing to evaluate the potency of RSR13-induced radiosensitization in combination with fractionated irradiation. Mice bearing SCCVII tumours were irradiated to 8 × 2.5 Gy over 4 days, 20 min after RSR13 injection. Four alternative schedules of combined RSR13 administration and irradiation were compared (see Figure 1). The survival ratio (SR) is defined as the SF obtained under a given experimental condition divided by the SF under control conditions, as noted in the legend in Table 2. The radiosensitizing effect of RSR13 is demonstrated by SR values ranging from 0.58 to 0.93 relative to 1.0 for radiation alone (Table 2). All three schedules involving RSR13 administration on consecutive days were significantly different from the control saline injections by standard unpaired *t*-test. The results of

© Cancer Research Campaign 1999

**Table 1** Results of FSaII tumour single-exposure cell survival curves: RSR13 dose–response and effects of air vs. carbogen breathing

| Inspiratory gas | Air | | | Carbogen | | |
|---|---|---|---|---|---|---|
| RSR13 dose (mg kg–1) | 0 | 100 | 300 | 0 | 100 | 300 |
| ER | 1.0 | 1.27 | 0.62 | 1.24 | 1.35 | 1.75 |
| $SF_{15}$ | 0.069 | 0.031 | 0.160 | 0.026 | 0.021 | 0.005 |

ER = enhancement ratios (see Materials and methods section); $SF_{15}$ = surviving fraction after 15 Gy.

specialized tests for multiple comparisons are also included in Table 2. The Newman–Kuels test confirms the observations of the *t*-test, indicating that the results are valid and do not reflect random differences that can result from comparisons of numerous data sets. The more stringent Tukey test, which produces broader confidence intervals, was also used in an effort to differentiate between relative radiosensitizing effects of the different schedules. As indicated in Table 2, the most significant difference occurred between the controls and the schedule involving RSR13 administration on days 1 and 2.

### DISCUSSION

The present work has demonstrated that RSR13, a synthetic allosteric effector of Hb, can emulate the function of the physiological modifier 2,3-DPG. The multipoint tonometry on whole-blood samples demonstrates the potent and rapid impact of RSR13 on the OEC in vivo, reflected in a *p*50 increase approaching 50 mmHg within 20 min after maximum RSR13 doses of 300 mg $kg^{-1}$. This reduction in Hb–$O_2$ affinity has been demonstrated to result in improved oxygen delivery to normal tissues (Khandelwal et al, 1993) and malignant tumours (Teicher et al, 1996). The increased tumour oxygenation led to tumour radiosensitization which was dependent upon the magnitude of increase in *p*50 and the atmospheric $pO_2$, as illustrated in Figures 3 and 4. Under conditions of air breathing, enhanced radiosensitivity was observed for the FSaII fibrosarcoma after an RSR13 dose of 100 mg $kg^{-1}$, whereas a dose of 300 mg $kg^{-1}$ had a radioprotective effect. This pattern was reversed by carbogen breathing. The radiosensitizing effect of carbogen alone was significantly amplified by the administration of RSR13 at doses of 100 or 300 mg $kg^{-1}$.

The multifactorial aetiology of tumour hypoxia makes strategies to overcome it challenging. Factors contributing to tumour hypoxia are increased oxygen consumption as a result of unrestricted cell proliferation (Nordsmark et al, 1996*b*) and inefficient oxygen delivery as a consequence of widely variable interstitial pressure gradients and irregular microvessel distribution (Jain, 1988). Different attempts to increase oxygen delivery to tumours have included the use of blood flow modifiers (Horsman et al, 1991) and the use of hyperbaric oxygen (Fletcher et al, 1977; Ward and Dixon, 1979; Dische, 1983; Brady et al, 1981). Neither approach has demonstrated consistent success in clinical trials to warrant therapeutic application outside investigative protocols. The radiosensitizing effect demonstrated for the allosteric effector of Hb, RSR13, supports earlier experimental work on 2,3-DPG (Siemann and Macler, 1986) and establishes the pharmacological manipulation of Hb–$O_2$ affinity as a new basis for increased oxygen delivery to tissues. The preclinical results on two tumour systems in single- and multidose radiation administration schedules demonstrate significant promise for RSR13 as a sensitizer of therapeutic irradiation.

There is uncertainty regarding the relative contributions to overall tumour hypoxia of diffusion-limited oxygen gradients and longitudinal oxygen gradients from the arterial to the venous capillary bed. Although the factors limiting oxygen diffusion perpendicular to the direction of blood flow through microvasculature have been recognized and investigated, only very recently has attention been directed to the large gradient of $pO_2$ from the arterial to the arteriolar circulation, a potential decrease of 50–70% in normal and tumour tissues (Dewhirst et al, 1996). Given the mechanism of decreasing Hb–$O_2$ affinity by RSR13, it is likely that the drug will be most influential at the arteriole–capillary interface by facilitating release of oxygen from partially depleted Hb. Because there is a steep gradient of $pO_2$ from the arterial to the arteriolar circulation, it is essential to have maximal oxygen loading within the pulmonary capillary circulation. With extreme increases in *p*50, such as those achieved by an RSR13 dose of 300 mg $kg^{-1}$, air breathing exerted a radioprotective effect due to insufficient saturation of Hb with oxygen in the pulmonary venous circulation. Consequently, peripheral delivery of oxygen was compromised, exacerbating the degree of tumour hypoxia. Because the high inspiratory $pO_2$ values associated with carbogen breathing can provide near-complete saturation of Hb (Figure 1B), the potential advantages of reduced Hb–$O_2$ affinity can be exploited to enhance radiosensitivity in tumour tissues.

Although the present experiments with FSaII tumours were not designed to estimate the hypoxic fraction, it is possible to estimate the change in the hypoxic fraction in these tumours from the clonogenic survival data. In other studies, the fraction of radiobiologically hypoxic tumour cells has been determined by comparing ex vivo survival data after in vivo irradiation of tumours under

**Table 2** The effect of RSR13 administration on the survival of irradiated SCCVII tumour cells receiving a course of fractionated radiation doses (8 × 2.5 Gy in 4 days)

| Treatment/day(s) | Number of animals | SF | SR | *P*-value (*t*-test) | *P*-value (Newman–Kuels) | *P*-value (Tukey) |
|---|---|---|---|---|---|---|
| Saline/1–4 | 22 | 0.0564 | 1.00 | – | – | – |
| RSR13/1,2 | 21 | 0.0326 | 0.58 | 0.0003 | <0.01 | <0.01 |
| RSR13/3,4 | 23 | 0.0362 | 0.64 | 0.0031 | <0.05 | <0.05 |
| RSR13/1–4 | 20 | 0.0378 | 0.67 | 0.0230 | <0.05 | NS |
| RSR13/1,4 | 14 | 0.0526 | 0.93 | NS | NS | NS |

SF = surviving fraction; SR = survival ratio = $SF_{RSR13\ treatment}/SF_{saline\ control}$; NS = not significant.

 © Cancer Research Campaign 1999

non-perturbed conditions and conditions of complete hypoxia typically achieved by clamping the arterial supply (Moulder and Rockwell, 1984). In the dose range above 10 Gy, cell survival is expected to be almost completely dependent on the presence of hypoxic cells; the fraction of hypoxic cells can be estimated by determining the vertical distance of the parallel lines fitted to data plotted on a semilog graph. The curves in Figure 3 fitted by linear regression are not parallel because they include data from 5- and 8-Gy dose points, and a substantial proportion of fully oxygenated cells could survive this dose. However, listed in Table 1 are the surviving fraction (SF) values obtained after 15 Gy, at which dose the number of surviving cells should predominantly reflect the initial burden of hypoxic cells. Comparison of the SF under control conditions with those obtained under the various experimental conditions reveals that air breathing with 100 mg $kg^{-1}$ RSR13 reduces the SF to 0.031 from the control value of 0.069, consistent with a 55% reduction of hypoxic cells. Likewise, carbogen breathing with 100 mg $kg^{-1}$ or 300 mg $kg^{-1}$ RSR13 reduces the hypoxic fraction by approximately 70% and 93% respectively.

The other key aspect of RSR13-mediated radiosensitization examined in this study was the effect of RSR13 administration during a course of repeated radiation exposures. Using the SCCVII tumour model, RSR13 given at a dose of 300 mg $kg^{-1}$ with carbogen breathing during at least 2 consecutive days of the irradiation scheme (see Figure 1) resulted in significant radiosensitization relative to radiation alone, providing a 33–42% enhancement of cytotoxicity (Table 2). Although others have used SCCVII tumours to evaluate the relative timing of radiation and other hypoxic cell radiosensitizers (Brown and Lemmon, 1990), the tumour model has not been applied previously to agents that enhance oxygen delivery. The results from our present study suggest that the radiosensitization of RSR13 is greatest when administered during the first half of the treatment course. These results are compatible with changes in tumour oxygenation expected to occur after irradiation alone and have been previously described as radiation-induced reoxygenation (Van Putten and Kallman, 1968). However, the pattern of reoxygenation can be variable (Goda et al, 1995), and further estimation of radiation-induced changes of tumour oxygenation under the present conditions of repeated radiation exposures will be required.

It is also noteworthy that RSR13 administered during the second half of the course of repeated radiation exposures still provides a significant 36% radiosensitization, suggesting the continued presence of hypoxic cells. One contributing factor to these findings may be the relatively short course of simulated fractionated irradiation over only 4 days, which is unlikely to allow for effective reoxygenation. Therefore, findings from the present study are currently being extended using a more protracted, once-daily irradiation schedule. Such studies are important because of their bearing on the design of clinical trials attempting to demonstrate a benefit for combined administration of RSR13 and radiation relative to irradiation alone. A phase IB tolerance study in human cancer patients has established that repeated daily 100 mg $kg^{-1}$ doses of RSR13 may be safely administered intravenously during a 2-week course of fractionated radiotherapy and that the agent exerts measurable increases in $p50$ (Kavanagh et al, 1997). However, for a more protracted course of radiotherapy in a potentially curative situation, it might be desirable to use RSR13 for only a portion of the entire treatment course; consequently, it is important to identify the portion during which RSR13 will provide the greatest benefit. Our observation of schedule-dependent variability in the magnitude of RSR13-mediated radiosensitization suggests that additional studies are needed to define the schedule of RSR13/radiation co-administration that yields maximum radiosensitization.

In summary, RSR13 enhances the toxicity of radiation towards two hypoxic tumour systems in vivo after single or repeated radiation exposures. Our results illustrate the importance of Hb loading with oxygen for the highly effective allosteric effector of Hb, RSR13, and the degree of reduction of $Hb–O_2$ affinity for effective radiosensitization. Additionally, the present study shows that the scheduling of RSR13 with radiation affects the magnitude of RSR13-mediated radiosensitization. Combining RSR13 with radiation earlier in a course of fractionated irradiation might yield the most pronounced effect by diminishing the burden of hypoxic cells during subsequent radiation exposures.

### ACKNOWLEDGEMENTS

This work was supported by the grant IN-105U from the American Cancer Society, Allos Therapeutics grant, National Heart, Lung, and Blood Institute grant R01-HL-32793 (Donald Abraham), and the Department of Radiation Oncology Florence and Hyman Meyers Head and Neck Cancer Research Fund. We thank Dr JM Brown (Stanford University) for supplying the initial SCCVII tumour stock and Allos Therapeutics, Denver, CO, USA, for supplying RSR13. We also thank Drs Michael J Gerber, Robert Steffen and Steve Hoffman (Allos Therapeutics), and Jurgen Venitz (VCU Department of Pharmacy and Pharmaceutics) for their helpful suggestions during preparation of this manuscript.

### REFERENCES

Abraham DJ, Peascoe RA, Randad RS and Panikker J (1992*a*) X-ray diffraction study of di and tetra-ligated T-state hemoglobin from high salt crystals. *J Mol Biol* **227**: 480–492

Abraham DJ, Wireko FC, Randad RS, Poyart C, Kister J, Bohn BB, Liard JF and Kunert MP (1992*b*) Allosteric modifiers of hemoglobin: 2-[4-[[(3, 5-disubstituted anilino)carbonyl]methyl]phenoxy]-2-methylpropionic acid derivatives that lower the oxygen affinity of hemoglobin in red cell suspensions, in whole blood, and in vivo in rats. *Biochemistry* **31**: 9141–9149

Brady LW, Plenk HP, Hanley JA, Glassburn JR, Kramer S and Parker RG (1981) Hyperbaric oxygen for carcinoma of the cervix – stages IIB, IIIB and IVA: results of a randomized study by the Radiation Therapy Oncology Group. *Int J Radiat Oncol Biol Phys* **7**: 991–998

Brizel DM, Sibley GS, Prosnitz LR, Scher R and Dewhirst MW (1997) Tumor hypoxia adversely affects the prognosis of carcinoma of the head and neck. *Int J Radiat Oncol Biol Phys* **38**: 285–290

Brown JM and Lemmon MJ (1990) Potentiation by the hypoxic cytotoxin SR 4233 of cell killing produced by fractionated irradiation of mouse tumor. *Cancer Res* **50**: 7745–7749

Bush RS, Jenkin RDT, Allt WEC, Beale FA, Bean H, Dembo AJ and Pringle JF (1978) Definitive evidence for hypoxic cells influencing cure in cancer therapy. *Br J Cancer* **37** (suppl. III): 302–306

Dewhirst MW, Ong ET, Rosner GL, Rehmus SW, Shan S, Braun RD, Brizel DM and Secomb TW (1996) Arteriolar oxygenation in tumour and subcutaneous arterioles: effects of inspired air oxygen content. *Br J Cancer* **74** (suppl. XXVII): S241–S246

Dische S, Anderson PJ, Sealy R and Watson ER (1983) Carcinoma of the cervix – anaemia, radiotherapy and hyperbaric oxygen. *Br J Radiol* **56**: 251–255

Dorie MJ, Menke D and Brown JM (1993) Comparison of the enhancement of tumor responses to fractionated irradiation by SR 4233 (tirapazamine) and by nicotinamide with carbogen. *Int J Radiat Oncol Biol Phys* **28**: 145–150

Evans JC and Bergsjo P (1965) The influence of anaemia on the results of radiotherapy in carcinoma of the cervix. *Radiology* **84**: 709–717

© Cancer Research Campaign 1999

Fletcher GL, Lindberg RD, Caderao JB and Wharton JT (1977) Hyperbaric oxygen as a radiotherapeutic adjuvant in advanced carcinoma of the uterine cervix: preliminary results of a randomized trial. *Cancer* **39**: 617–623

Gatenby RA, Kessler HB, Rosenblaum JS, Coia LR, Moldofsky PJ, Hartz WH and Broder GJ (1988) Oxygen distribution in squamous cell carcinoma metastases and its relationship to outcome of radiation therapy. *Int J Radiat Oncol Biol Phys* **14**: 831–838

Gerweck LE, Koutcher JA, Zaidi ST and Seneviratne T (1992) Energy status in the murine FSaII and MCaIV tumors under aerobic and hypoxic conditions: an in-vivo and in-vitro analysis. *Int J Radiat Oncol Biol Phys* **23**: 557–561

Goda F, O'Hara JA, Rhodes ES, Liu KJ, Dunn JF, Bacic G and Swartz HM (1995) Changes of oxygen tension in experiment tumors after a single dose of X-ray irradiation. *Cancer Res* **55**: 2249–2252

Hall EJ (1994) *Radiobiology for the Radiologist*, 4th edn, pp. 133–152. JB Lippincott: Philadelphia

Hirst DG and Wood PJ (1987) The influence of haemoglobin affinity for oxygen on tumour radiosensitivity. *Br J Cancer* **55**: 487–491

Hirst DG and Wood PJ (1989) Chlorophenoxy acetic acid derivatives as hemoglobin modifiers and tumor radiosensitizers. *Int J Radiat Oncol Biol Phys* **16**: 1183–1186

Hirst DG, Wood PJ and Schwartz HC (1987) The modification of hemoglobin affinity for oxygen and tumor radiosensitivity by antilipidemic drugs. *Radiat Res* **112**: 164–172

Hockel M, Knoop C, Schlenge K, Vorndran B, Baubmann E, Mitze M, Knapstein PG and Vaupel P (1993) Intratumoral $pO_2$ predicts survival in advanced cancer of the uterine cervix. *Radiother Oncol* **26**: 43–50

Horsman MR, Chaplin DJ and Overgaard J (1991) The use of blood flow modifiers to improve the treatment response of solid tumors. *Radiother Oncol* **20** (suppl.): 47–52

Horsman MR, Khalil AA, Siemann DW, Grau C, Hill SA, Lynch EM, Chaplin DJ and Overgaard J (1994) Relationship between radiobiological hypoxia in tumors and electrode measurements of tumor oxygenation. *Int J Radiat Oncol Biol Phys* **29**: 439–442

Jain RK (1988) Determinants of tumor blood flow. *Cancer Res* **48**: 2641–2658

Kavanagh BD, Khandelwal SR, Schmidt-Ullrich RK, Roberts JD, Pearlman AD, Shaw EG, Venitz J and Gerber MJ (1997) A phase Ib study to evaluate repeated daily intravenous doses of RSR13 administered to cancer patients receiving concurrent radiation therapy (abstract). Proceedings of the 39th Annual ASTRO Meeting. *Int J Radiat Oncol Biol Phys* **39**: 330S

Khandelwal SR, Randad RS, Lin PS, Meng H, Pittman RN, Kontos HA, Choi SC, Abraham DJ and Schmidt-Ullrich R (1993) Enhanced oxygenation in vivo by allosteric inhibitors of hemoglobin saturation. *Am J Physiol* **265**: H1450–H1453

Khandelwal SR, Lin PS, Hall CE, Truong QT, Lu J, Laurent JJ, Joshi GS, Abraham DJ and Schmidt-Ullrich RK (1996) Increased radiation response of FsaII fibrosarcomas in C3H mice following administration of an allosteric effector of hemoglobin–oxygen affinity. *Radiat Oncol Invest* **4**: 51–59

Moulder JE and Rockwell S (1984) Hypoxic fractions of solid tumors: experimental techniques, methods of analysis, and a survey of existing data. *Int J Radiat Oncol Biol Phys* **10**: 695–712

Nordsmark M, Overgaard M and Overgaard J (1996*a*) Pretreatment oxygenation predicts radiation response in advanced squamous cell carcinoma of the head and neck. *Radiother Oncol* **41**: 31–39

Nordsmark M, Hoyer M, Keller J, Nielsen OS, Jensen OM and Overgaard J (1996*b*) The relationship between tumor oxygenation and cell proliferation in human soft tissue sarcomas. *Int J Radiat Oncol Biol Phys* **35**: 701–708

Okunieff PG, Koutcher JA, Gerweck L, McFarland E, Hitzig B, Urano M, Brady T, Nueringer L and Suit HD (1986) Tumor size dependent changes in a murine fibrosarcoma: use of in vivo $^{32}P$ NMR for non-invasive evaluation of tumor metabolic status. *Int J Radiat Oncol Biol Phys* **12**: 793–799

Overgaard J (1994) Clinical evaluation of nitroimidazoles as modifiers of hypoxia in solid tumors. *Oncol Res* **6**: 509–518

Overgaard J, Hansen HS, Andersen AP, Hjelm-Hansen M, Jorgensen K, Sandberg E, Berthelsen A, Hammer R and Pedersen M (1989) Misonidazole combined with split-course radiotherapy in the treatment of invasive carcinoma of larynx and pharynx: report from the DAHANCA 2 study. *Int J Radiat Oncol Biol Phys* **16**: 1065–1068

Randad RS, Mahran MA, Mehanna AS and Abraham DJ (1991) Allosteric modifiers of hemoglobin. 1. Design, synthesis, testing, and structure–allosteric activity relationship of novel hemoglobin oxygen affinity decreasing agents. *J Med Chem* **34**: 752–757

Rice L, Urano M and Suit HD (1980) The radiosensitivity of a murine fibrosarcoma as measured by three cell survival assays. *Br J Cancer* **41** (suppl. 4): 240–244

Siemann DW and Macler LM (1986) Tumor radiosensitization through reductions in hemoglobin affinity. *Int J Radiat Oncol Biol Phys* **12**: 1295–1297

Siemann DW, Hill RP, Bush RS and Chhabra P (1979) The in vivo radiation response of an experimental tumor: the effect of exposing tumor-bearing mice to a reduced oxygen environment prior to but not during irradiation. *Int J Radiat Oncol Biol Phys* **5**: 61–68

Teicher BA, Ara G, Emi Y, Kakeji Y, Ikebe M, Maehara Y and Buxton D (1996) RSR13: effects on tumor oxygenation and response to therapy. *Drug Dev Res* **38**: 1–11

Van Putten LM and Kallman RF (1968) Oxygenation status of a transplantable tumor during fractionated radiotherapy. *J Natl Cancer Inst* **40**: 441–451

Ward AJ and Dixon B (1979) Carcinoma of the cervix: results of a hyperbaric oxygen trial associated with the use of the cathetron. *Clin Radiol* **30**: 383–387

Wireko FC, Kellogg GE and Abraham DJ (1991) Allosteric modifiers of hemoglobin. 2. Crystallographically determined binding sites and hydrophobic binding/interaction analysis of novel hemoglobin oxygen effectors. *J Med Chem* **34**: 758–767

 *© Cancer Research Campaign 1999*
